# Professional Development for the Clinician‐Educator in Respiratory and Sleep Medicine

**DOI:** 10.1002/resp.70272

**Published:** 2026-06-10

**Authors:** Mark Lavercombe

**Affiliations:** ^1^ Department of Respiratory & Sleep Disorders Medicine Western Health Footscray Australia; ^2^ Department of Medical Education The University of Melbourne Melbourne Australia

**Keywords:** clinician‐educator, professional development, respiratory medicine, sleep medicine

Clinician‐educators are more than just doctors who teach; they are experts in their clinical practice, engaged with educational theory, advisors on teaching excellence, and contributors to educational scholarship [1]. Mastery of their dual roles requires the clinician‐educator to develop skills in instructional and curriculum design, adult learning theories, assessment and evaluation, feedback and remediation, professionalism, and the language and confidence to participate in scholarly conversations about education and lead educational teams [2]. These skills aren't innate; instead, they are cultivated through intentional development, active participation in professional learning, and reflective practice.

In this commentary, I will outline the specific challenges clinician‐educators face in their professional development and strategies they might choose as they aim to join the ranks of the experts they admire. I also reflect on formative experiences and opportunities that have shaped my journey as a clinician‐educator, as well as the structural changes required for our field to thrive. (Figure 1)

In 2013, a local educational leader asked my clinical head of department if she had any staff who might be interested in taking on some bedside teaching for medical students to cover another educator's leave. I had some time available and started the tutorials, only to find I genuinely enjoyed working with those students and helping them develop their skills. I had never really considered medical education as an option, but now I describe myself as a clinician‐educator without hesitation.

**FIGURE 1:**
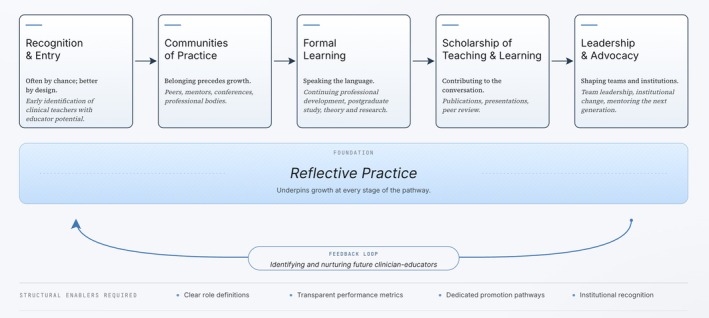
A framework for the clinician‐educator's developmental pathway. Progression through five stages—Recognition & Entry, Communities of Practice, Formal Learning, Scholarship of Teaching & Learning, and Leadership & Advocacy—is supported by ongoing reflective practice, and established clinician‐educators close the loop by identifying and nurturing the next generation. Structural reform of role definitions, performance evaluation, and promotion pathways is required to enable the journey. (Figure was conceptualised by the author, with its content, labels, and structure developed by the author, and the visual rendering produced with assistance from Claude Design.)

My start as an educator is not unique; many clinicians report similar experiences [3]. Since university‐based educators conduct only a minority of medical training and most occurs during clinical placements, a more proactive approach to recruiting and developing clinician‐educators could be beneficial [4]. Establishing a clear career pathway for clinician‐educators, with defined competencies and performance metrics, promoted from the early stages of medical training—similar to the way students interested in surgery might be identified and nurtured—would be an investment in future educational capacity.

An advantage that clinician‐educators enjoy is the authenticity that their clinical role ensures [5]. Learners feel confident that their instruction is grounded in active, ongoing medical practice. Further, clinician‐educators benefit from direct interactions that provide insight into the barriers and challenges junior clinicians face in their daily work. Credibility is enhanced when learners see their educators on the wards, in the clinics, or in the intensive care unit.

The role of clinician‐educator also presents significant challenges. To switch easily between their specialist roles, the practitioner must be an expert in their clinical field while building the knowledge, experience, and reputation needed to influence educational programme delivery and be recognised as an educator [6]. Achieving these objectives requires time, effort, and evidence of impact in both practice areas.

Balancing their commitments to teaching and professional development as educators with the demands of busy clinical service can be difficult, and many clinician‐educators find that their clinical work negatively impacts essential educational activities, such as producing educational scholarship [7]. While some authors recommend dedicated time for scholarly activities (e.g., one half‐day per week), managing dual roles and their conflicting obligations remains challenging [8].

Finally, a lack of clear role definitions, unclear performance evaluation metrics, and a substantial body of literature describing challenges in achieving academic promotion mean that clinician‐educators face real barriers to their professional and career development [6, 9, 10]. Overcoming these barriers requires tenacity and a strategic approach that enables the educator to address gaps in their knowledge and experience while building an academic profile.

As I developed my interest in education, I began to feel even lonelier in my clinical practice. A chance conversation at the CHEST 2016 conference with an experienced clinician‐educator led to an invitation to speak on a panel the following year, which in turn led to an opportunity to join the educator development subcommittee. To my shock, I had found people with the same professional interests as me who embraced me as a peer. My experience since then has been transformational.

If you don't have a local mentor or peer group, get involved in professional bodies. Major respiratory medicine societies often have sections dedicated to education and educator development. Participants in these groups tend to share your interests, as you practise in the same medical specialty and focus on education [11]. I now belong to an international community of practice in respiratory medicine education, where I have made friends, pursued scholarship, published research, and strengthened my professional identity as an educator [12]. These experiences gave me the confidence to volunteer with health professional education societies such as ANZAHPE (Australian & New Zealand Association for Health Professional Educators) and AMEE (The International Association for Health Professions Education)—something I couldn't have imagined before being nurtured by my clinician‐educator colleagues.

For clinician‐educators in our region, the Asia Pacific Medical Education Conference (APMEC) is held annually and serves as an important venue for connecting with peers across the region. Coordinated by the Centre for Medical Education at the National University of Singapore, it has been held in Singapore and China in recent years, with Japan set to host in 2027 [13]. The ANZAHPE and AMEE conferences—now explicitly internationally oriented—both draw participation from clinician‐educators across South‐East Asia and beyond, providing opportunities to build collegial networks that extend beyond one's home institution.

In addition to conferences, professional bodies often run mentorship programmes that can assist junior educators in finding someone who offers critical advice and support from a perspective outside their local institution(s). This can be valuable in pinpointing where a clinician‐educator needs to develop their skills, knowledge or experience, and in managing the frustrations of working with colleagues who don't truly understand your role. My experience as a mentee brought me real clarity about what I hoped to achieve as a clinician‐educator and how to take the next steps [14].

Underpinning growth as a mentee is reflective practice. Making time to reflect on your successes, and especially on things that didn't go as planned and the gaps they might highlight, can be crucial in ongoing professional development [15]. Beyond mentoring, the evidence shows that learning in continuing professional development programmes is greatly improved by including opportunities for reflection, making this a best practice in their design. Reflection is a key element of the Royal Australasian College of Physicians' ‘MyCPD’ programme for this reason [16].

After several years working as a clinical teacher in a medical degree, I enrolled in a postgraduate programme in clinical education. Interacting with peers from a broad range of health professions and the outstanding array of faculty educators helped me recognise my knowledge gaps and understand the scope of educational theory and educator responsibilities. This humbling yet inspiring experience provided a roadmap for my ongoing professional development as a clinician‐educator.

As a junior educator, you may not yet grasp the breadth of knowledge and literature in health professional education, and formal programmes can help provide that insight [9, 17]. Although not every clinician‐educator needs a higher degree in education, to operate effectively within academic institutions and influence change, you need credibility. Formal study and research in education will give your opinion more weight when working with curriculum developers or programme designers [17]. Speaking the language can be just as important as demonstrating long‐term commitment to your craft.

Even if you decide not to enrol in a higher degree, actively choose to participate in at least one continuing professional development programme each year that enhances your *educational* skills or knowledge. The literature on the specific competencies required for success as a clinician‐educator can guide you in planning your activities [2]. Ensure you focus on leadership skill development as an ongoing priority, as senior clinician‐educators can be a critical component of an academic department's success [18]. As a future leader, the more you have learned and practised in this area, the more effective you will be in advocating for your junior clinician‐educator colleagues.

Crucially, you should start developing your scholarship of teaching and learning as early as possible. Contributing to the scholarly conversation is a key way to consolidate your professional interests, enhance your academic reputation, and invite collaborations with like‐minded educators [19]. Submitting novel educational materials for peer review to platforms such as MedEdPublish can be a way to get started [20]. Conference presentations and academic publications in your clinical area are often more achievable than aiming for one of the major medical education journals, at least until you've built a body of educational work.

I was repeatedly told not to apply for academic promotion and that it is difficult for a clinician‐educator to meet the required standards. After several years of delay, I decided to apply, but found the process was heavily tailored to researchers, and the feedback I received along the way led to prolonged self‐doubt, a lack of motivation, and decreased job satisfaction. If my colleagues didn't value my work, should I persevere?

Applying for academic promotion *should* be challenging; you need to demonstrate that your contributions in education, research, service, and leadership meet the required standards [21]. The lack of transparent metrics to evaluate educational impact remains a problem that promotion committees have yet to resolve, and assessing your contributions across two domains of work makes the committee's task even more difficult.

Inequity in promotion and advancement for clinician‐educators has been recognised for decades. Beasley's 1997 survey of 115 medical schools across the United States and Canada revealed that committees relied on teaching awards, peer and learner evaluations, and teaching portfolios to assess educational impact [22]. The survey highlighted that contributions to educational programme design and delivery were not being considered. A later comparison of department chairs and promotion committee chairs found significant differences in how they weighed clinician‐educator contributions [10]. Committee chairs placed greater emphasis on journal prestige and external grant support, and less on programme administration and leadership qualities (confirming Beasley's findings). This exemplifies the dilemma facing an individual clinician‐educator working towards promotion: should you focus more on the work activities your department values or on those that will enhance your advancement opportunities?

Calls for separate performance evaluation criteria and dedicated pathways for clinician‐educators have drawn mixed responses. One recent paper proposed expanding the definition of educational scholarship and measuring it more broadly than by publication metrics; expanding how expertise is measured to rely less on learner feedback, given its documented limitations; and ensuring that clinician‐educators are appointed to promotion committees [23].

A 2026 position paper from Medical Deans Australia and New Zealand argues for systemic change in the identification, recruitment, nurturing, retention, and recognition of clinician‐educators, as part of a national framework with clear role definitions and professional standards [24]. Despite decades of awareness of the specific challenges clinician‐educators face in their dual roles, in having their performance evaluated, and in achieving academic advancement, this paper reminds us how much work remains to be done.

As a doctor in training, I learned a lot about the practice of medicine, and a dominant theme was my desire for my juniors to have a better experience than I did. As a mid‐career clinician‐educator, I now feel a responsibility to nurture junior colleagues, support their growth in this demanding role, and advocate for a more transparent and equitable pathway.

If you are just starting out as a clinician‐educator, this piece should serve as a guide as you begin your journey. Once you become more established, however, you might be the standard‐bearer for the role at your institution. Ensure you have your supervisors' support, ask what your organisation really needs, and consider how you might tailor your ongoing development to meet those gaps. Partnerships with colleagues or professional societies at the national or international level will be critical to achieving the authority required to lead change. Never forget to ask your learners about their experiences to ensure you're calibrated to meet their needs, and remember to actively identify juniors who might consider a role in medical education.

We deserve better than serendipity. As a craft group, we clinician‐educators must actively pursue the structural and systemic reforms called for in the literature so that future educators may choose this path without hesitation.

## Funding

The author has nothing to report.

## Conflicts of Interest

Mark Lavercombe is an Editorial Board member of Respirology and was excluded from all editorial decision‐making related to the acceptance of this article for publication.

## Data Availability

Data sharing not applicable to this article as no datasets were generated or analysed during the current study.
